# Teaching Strategies for Interdisciplinary Collaboration within Gerontology Education

**DOI:** 10.1093/geroni/igaf122.3490

**Published:** 2025-12-31

**Authors:** Elaine Jurkowski

**Affiliations:** Southern Illinois University, Carbondale, Illinois, United States

## Abstract

A critical area within gerontological education and medical/health/behavioral professions is to educate and train students for competence within interprofessional collaboration. Based on the experiential learning theory (Kolb & Kolb, 2017) and drawing from the IPCE model (Interprofessional Education Collaborative, 2023), this study has creatively designed a training/education model for students within gerontological fields of practice to enhance their ability to understand the concept of working collaboratively across disciplines. Students were placed in teams utilizing one of at least three models of experiential education and worked across teams with real patient/consumer consultations/case management strategies. The goal was to achieve both patient/consumer outcomes and learn about professional practice with people growing older using an interdisciplinary collaborative approach. Three specific strategies used include “Student Hotspotting”, an Interdisciplinary Day (IPE), and a collaborative teaching clinic. The Student Hotspotting approach, modelled after the Camden Coalitions’ program was an academic year long experience working with a older person with complex care needs. The IPE day focused on a case study approach and guest presenters via a team-based approach with health-oriented disciplines working as a team to identify a case management plan. The Teaching Clinic brought together medical, social work, nursing, physician assistant and nutrition students together in an outpatient clinic setting to address patients with complex care needs and aging related health conditions. All three strategies examined care through the lens of Social Determinants of Health. Evaluation outcomes presented positive outcomes and benefits to the students’ learning outcomes and patient outcomes.

